# Angiopoietin-2 is associated with capillary leak and predicts complications after cardiac surgery

**DOI:** 10.1186/s13613-023-01165-2

**Published:** 2023-08-08

**Authors:** Jakob Wollborn, Zilu Zhang, Julie Gaa, Moritz Gentner, Christian Hausmann, Felix Saenger, Karina Weise, Samuel Justice, Jean-Luca Funk, Hans Felix Staehle, Marie Thomas, Raphael R. Bruno, Babak Saravi, Jan O. Friess, Markus Marx, Hartmut Buerkle, Georg Trummer, Jochen D. Muehlschlegel, Daniel Reker, Ulrich Goebel, Felix Ulbrich

**Affiliations:** 1 Department of Anesthesiology, Perioperative and Pain Medicine, Brigham and Women’s Hospital, Harvard Medical School, 75 Francis Street, Boston, MA 02115 USA; 2 Department of Anesthesiology and Critical Care, Medical Center, University of Freiburg, Breisgau, Germany; 3 Department of Biomedical Engineering, Duke University, Durham, NC USA; 4 Department of Cardiovascular Surgery, Medical Center, University of Freiburg, Freiburg, Germany; 5 Department of Cardiology, Pulmonology and Vascular Medicine, Medical Faculty, Heinrich-Heine-University Duesseldorf, University Hospital Duesseldorf, Duesseldorf, Germany; 6 Department of Anesthesiology, Critical Care and Pain Medicine, Boston Children’s Hospital, Harvard Medical School, Boston, USA; 7 Department of Anesthesiology and Critical Care, St. Franziskus-Hospital, Muenster, Germany; 8 Faculty of Medicine, University of Freiburg, Freiburg, Germany

**Keywords:** Cardiac surgery, Capillary leak syndrome, Critical care, Fluid balance, Endothelial permeability, Angiopoietin-2, Acute kidney injury

## Abstract

**Background:**

Patients undergoing cardiac surgery are prone to numerous complications. Increased vascular permeability may be associated with morbidity and mortality due to hemodynamic instability, fluid overload, and edema formation. We hypothesized that markers of endothelial injury and inflammation are associated with capillary leak, ultimately increasing the risk of postoperative complications.

**Methods:**

In this prospective, observational, multidisciplinary cohort study at our tertiary academic medical center, we recruited 405 cardiac surgery patients. Patients were assessed daily using body impedance electrical analysis, ultrasound, sublingual intravital microscopy, and analysis of serum biomarkers. Multivariable models, as well as machine learning, were used to study the association of angiopoietin-2 with extracellular water as well as common complications after cardiac surgery.

**Results:**

The majority of patients underwent coronary artery bypass grafting, valvular, or aortic surgeries. Across the groups, extracellular water increased postoperatively (20 ± 6 preoperatively to 29 ± 7L on postoperative day 2; *P* < 0.001). Concomitantly, the levels of the biomarker angiopoietin-2 rose, showing a strong correlation based on the time points of measurements (r = 0.959, *P* = 0.041). Inflammatory (IL-6, IL-8, CRP) and endothelial biomarkers (VE-Cadherin, syndecan-1, ICAM-1) suggestive of capillary leak were increased. After controlling for common risk factors of edema formation, we found that an increase of 1 ng/mL in angiopoietin-2 was associated with a 0.24L increase in extracellular water (*P* < 0.001). Angiopoietin-2 showed increased odds for the development of acute kidney injury (OR 1.095 [95% CI 1.032, 1.169]; *P* = 0.004) and was furthermore associated with delayed extubation, longer time in the ICU, and a higher chance of prolonged dependence on vasoactive medication. Machine learning predicted postoperative complications when capillary leak was added to standard risk factors.

**Conclusions:**

Capillary leak and subsequent edema formation are relevant problems after cardiac surgery. Levels of angiopoietin-2 in combination with extracellular water show promising potential to predict postoperative complications after cardiac surgery.

*Trial registration number*: German Clinical Trials Registry (DRKS No. 00017057), Date of registration 05/04/2019, www.drks.de

**Graphical Abstract:**

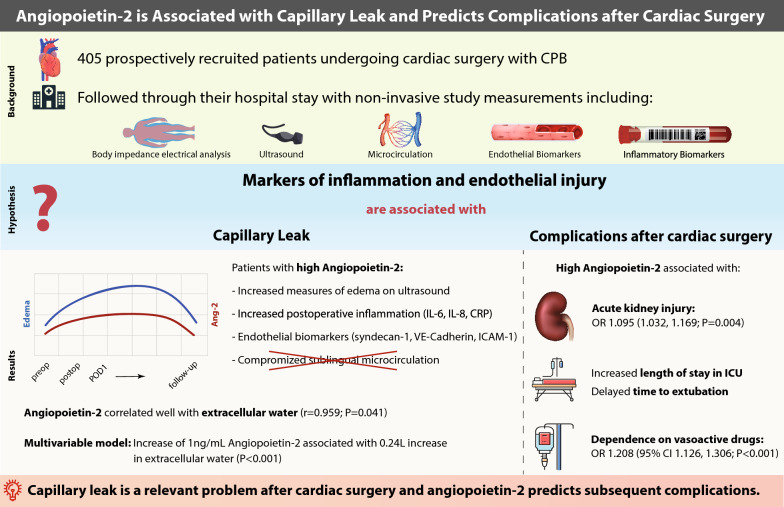

**Supplementary Information:**

The online version contains supplementary material available at 10.1186/s13613-023-01165-2.

## Introduction

Patients undergoing cardiac surgery are prone to numerous complications which may contribute to high morbidity and mortality [1]. It has previously been established that surgery with the use of cardiopulmonary bypass (CPB) induces a profound pro-inflammatory response leading to vascular injury which is further exacerbated by altered flow characteristics and shear stress on CPB [2]. Among inflammation-related complications, an increase in vascular permeability is of particular interest as it can lead to fluid extravasation, volume overload, and edema formation. This phenomenon is often termed Capillary Leak Syndrome (CLS) and, aside from cardiac surgery, frequently occurs in sepsis [3, 4], anaphylaxis [5], and thermal injury [6]. Fluid administration and vasoactive drugs may ultimately become necessary to stabilize hemodynamics. Up-to-date, substantial clinical data on cardiac surgery and CLS are missing. Despite the lack of widely accepted diagnostic criteria for CLS, elevated pro-inflammatory cytokine profiles are associated with adverse outcomes after cardiac surgery [7], and cytokine levels tend to be higher in non-survivors of critical illness [8]. Importantly, a positive fluid balance reflects an independent risk factor for death in patients after cardiac surgery [9].

Angiopoietin-2 (Ang-2) was previously shown to play an important role in the regulation of the vascular barrier. In a combined in-vitro and in-vivo study examining serum from 28 adult patients after coronary artery bypass grafting (CABG), increased in-vitro permeability in cultured endothelial cells was associated with an imbalance of angiopoietin-1 and -2 (Ang-1/-2) [10]. Our group recently substantiated that Ang-2 holds promising potential to identify CLS in a heterogeneous cohort of critically ill patients [4]. Here, CLS was associated with an increase in 30-day mortality and revealed distinct characteristics in its biomarker profiles.

Currently, the consequences of CLS and complications are insufficiently understood in cardiac surgery. In this prospective study, we hypothesized that Ang-2 levels are linked with markers of vascular permeability and edema formation in patients undergoing cardiac surgery patients, while an increase in serum Ang-2 consecutively leads to postoperative complications.

## Methods

### Design and setting

**Fig. 1:**
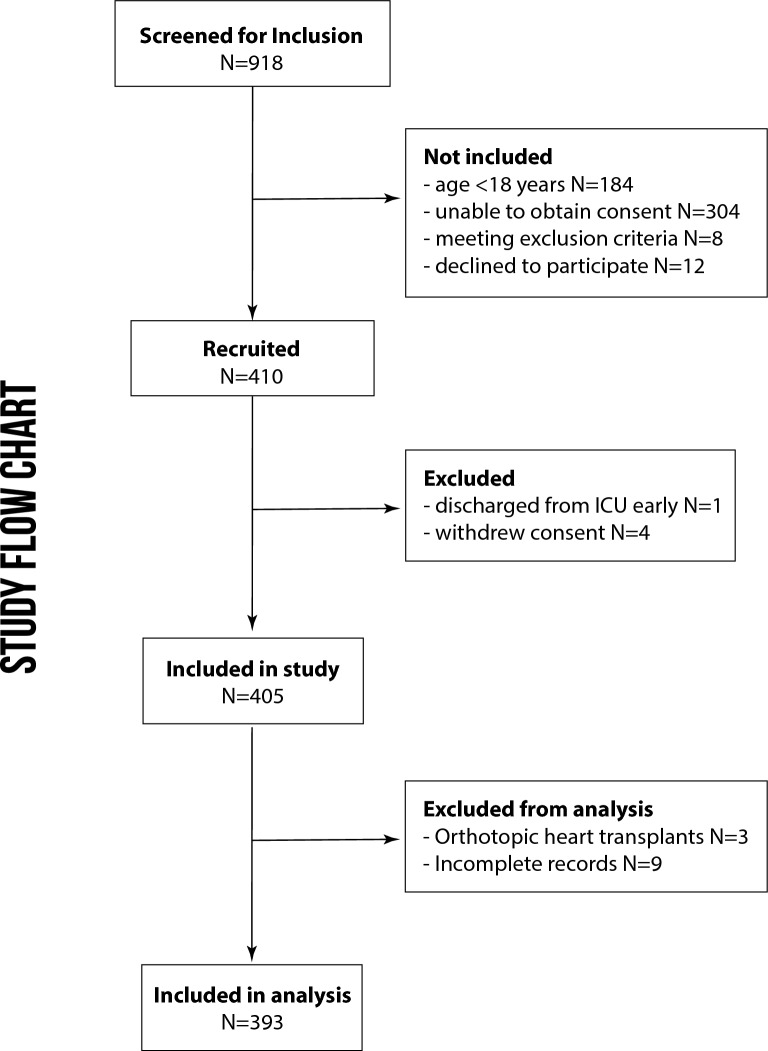
CONSORT study flow chart

The study was designed in a prospective, multidisciplinary, observational approach. Reporting complied with the STROBE (Strengthening the Reporting of Observational Studies in Epidemiology) statement [11]. Patients were recruited prior to their cardiovascular surgery and were followed postoperatively in the intensive care units at the Medical Center of the University of Freiburg, Germany from May 2019 to October 2020. The study was approved by the institutional review board (Freiburg, EK-Nr. 405/18) and registered (DRKS No. 00017057). All patients, their respective next-of-kin, or legal guardian had to provide informed consent for study participation. 918 patients were screened for study inclusion (see Fig. 1). If patients were still hospitalized seven days after discharge from the ICU, they were included in a 7-day follow-up.

### Study population

We assessed the eligibility of all adult patients scheduled for cardiovascular surgery with the anticipated use of CPB at our institution. Inclusion criteria were defined as planned cardiovascular surgery with the use of CPB, age ≥ 18 years, and informed consent. Patients were excluded if one or more of the following criteria applied: Refusal to participate, infection with HIV, viral hepatitis, idiopathic capillary leak syndrome (“Clarkson disease”), hereditary C1-esterase deficiency, recurrent angioedema, pre-existing chronic kidney failure requiring dialysis, pre-existing hepatic impairment with a MELD score ≥ 20 and participation in clinical trials.

### Clinical care

Routine clinical care was unchanged during the study. In brief, anesthesia was induced at the discretion of the responsible anesthesiologist and was maintained using a balanced technique with sevoflurane and sufentanil, while a propofol infusion was initiated prior to transport to the ICU. For CPB, customary circuits using cannulas, a roller head, and membrane oxygenators (Stöckert, Sorin, Germany) were used. After completion of the surgery, patients were admitted to the intensive care unit (ICU). Sedation was stopped when deemed safe by the ICU team, and extubation was then pursued in a timely manner. Administration of vasoactive and inotropic medication was at the discretion of the primary team and guided by invasive as well as non-invasive tools of hemodynamic monitoring. The decision to administer fluid was based on all available variables. This included the clinical examination, a positive fluid challenge or passive leg raise test, echocardiographic assessment, as well as invasive and non-invasive measurements, when deemed appropriate. Of note, only balanced crystalloid solutions were used for fluid resuscitation in our patients.

### Study measurements

The preoperative patient measurements were obtained after induction of general anesthesia with the postoperative measurement after completion of surgery. For every consecutive day in the ICU, patients were assessed daily after morning rounds until ICU discharge. If still hospitalized one week after ICU discharge, a 7-day follow-up (7FU) including study measurements was performed. A blood sample was obtained at every measurement timepoint. Patients’ electronical medical records (EMRs) were followed. For insight into body and fluid composition, body impedance electrical analysis (BIA) was used at every study measurement as previously described (Nutriguard-MS, Data Input GmbH, Poecking, Germany) [4]. In brief, the measurements were performed in a hand-to-foot approach with the patient supine. Data were analyzed using the NutriPlus software (Data Input GmbH, Poecking, Germany). The principle of BIA lies in measuring body’s impedance after an electrical current is sent through the body (tissue presents various levels of resistance) [12]. For this technique, non-invasive electrodes are applied to the patient’s skin. The phase angle is a raw measurement by BIA showing the relationship between reactance and resistance of tissue. Various other parameters can then be derived and calculated from the measurements (e.g., extracellular water). Standardized views on ultrasound were used to quantify tissue echogenicity as previously described [4]. Using incident-darkfield imaging with the CytoCam™ system (Braedius, Huizen, The Netherlands), sublingual microcirculation was assessed. Image acquisition, analysis of the microcirculatory sequences, and calculation of microcirculatory flow-derived variables was performed in accordance with current consensus guidelines [13]. Five different sublingual regions were assessed for every patient at every measurement timepoint. Video analysis was performed according to the consensus recommendation by five trained providers to gain insight into convective and diffusive flow properties. Patients’ serum samples were analyzed for cytokine levels, Angiopoietin-2, sVE-Cadherin, sICAM-1, and syndecan-1 (please see Additional file 1 for more detail). Available patient data such as demographics, medication, and laboratory values were obtained from the EMR.

### Statistical analysis

Patients were categorized in surgical groups. Patients undergoing combined interventions such as valve and revascularization surgery were grouped with the valvular surgery patients (due to the extent of the surgery and the necessity of a cardiotomy). Patients having combined aortic surgery with valve and/or CABG surgery were grouped with the aortic surgery patients. Due to a limited number of orthotopic heart transplants (*N* = 3), these patients were excluded from our analysis. Nine patients had incomplete records in our dataset and thus had to be excluded. The group of “other” surgeries comprised interventions such as ASD closures, pulmonic, or singular tricuspid valve intervention.

For detailed description of the statistics, please see the Additional file 1 section. To assess the association between Ang-2 and the primary outcome of extracellular water (ECW), generalized estimating equations (GEE) models with an identity link function and an exchangeable correlation structure were used to account for repeated measurements on patients throughout the perioperative period. We used a multivariable model to adjust for confounders (see Additional file 1). A similar model was fit for the association of Ang-2 with the P/F-ratio [14]. Logistic regression was employed to model the relationship between Ang-2 and the secondary outcomes of AKI and continuous dependence on vasoactive medication [15]. The association between Ang-2 and the secondary outcomes of time to ICU discharge and time to extubation were assessed by means of survival analysis and Cox proportional hazards models [16, 17].

### Machine learning

Machine learning was used to refine post-operative outcome prediction using data that were collected before or immediately after surgery. All patients were described using risk factors (see multivariable analyses, Additional file 1). Additionally, we created a second, augmented feature set that included the same features but also added absolute values, differences and relative ratios of accessible body impedance electrical analysis measurements and serum Ang-2 levels before and after the surgery. Raw data were cleaned and preprocessed prior to machine learning modeling using Python (V3.8, Python Software Foundation, USA). Specifically, patients who did not contain all features were excluded from the analysis. Mean value and standard deviation were calculated for every variable in the training data and used to standardize the training and test data (Z score transformation). Seven classifiers available in scikit-learn library (V1.0.2) were implemented with optimized hyperparameters, namely Gaussian Naïve Bayes (var_smoothing 1e-07), K-Nearest Neighbors Classifier (6 neighbors), Decision Tree (entropy, best split), Random Forest (100 trees, entropy), Linear Support Vector Machine (l2 penalty, squared hinge), AdaBoost Classifier (50 estimators, learning rate 0.25), and Neural Networks (multilayer perceptron, 3 hidden layers with 50 neurons per layer). Hyperparameters not listed were set to default. In addition to standard ten-fold cross validation, we also performed ten-fold stratified cross validation, leave-one-out cross validation to test every patient individually and leave-one-group-out cross validation on surgery type (see Additional file 9: Fig. S2). All experiments were repeated ten times to determine their variability and enable statistical analysis. Model performance was evaluated using area under the Receiver Operating Characteristic (ROC-AUC), Matthews’ correlation coefficient (MCC), Cohen’s κ coefficient, and balanced accuracy. Wilcoxon rank-sum or Mann–Whitney U test were used to assess statistical significance for different performance metrics.

## Results

918 individuals undergoing cardiac surgery with CPB at our institution were assessed for study inclusions. After the recruitment process and after excluding patients according to pre-defined criteria, records from 393 patients were analyzed (see Fig. 1).

Most patients underwent CABG, valvular, or major aortic procedures (see Table 1). The mean age in our cohort was 63 ± 14 with 75% males. 26 patients (7%) died within the observational time of 30 days. Patients with left ventricular assist device (LVAD) placement stayed in the ICU for 9 (6–10) days, while the general cohort had a median length of stay (LOS) of 3 (1–5) days. The median CPB time was 135 (105–165) minutes. In 45 patients (52%) undergoing aortic surgeries, deep hypothermic cardiac arrest (DHCA) was used. Postoperative extracorporeal membrane oxygenation (ECMO) was used in 15 patients (4%). 158 patients (40%) developed postoperative acute kidney injury (AKI). 129 (33%) patients presented with KDIGO stage I, 19 (5%) patients had KDIGO stage II, and 10 (3%) patients had KDIGO stage 3 AKI. Renal replacement therapy was necessary in 14 (4%) patients. 43 (11%) of patients had postoperative low-cardiac output syndrome (LCOS). For additional patient characteristics and echocardiographic assessment, see Additional file 2: Table S1 and Additional file 3: Table S2.

**Table 1:** Patient demographics and data on surgical characteristics and complications

	All patients (*N* = 393)	CABG (*N* = 100)	AV (*N* = 63)	MV (*N* = 52)	Multivalve (*N* = 32)	Aorta (*N* = 86)	LVAD (*N* = 21)	Others (*N* = 39)
General characteristics								
Age (mean ± SD)	63 ± 14	67 ± 11	66 ± 12	60 ± 10	69 ± 12	63 ± 14	57 ± 15	51 ± 17
Male (%)	295 (75%)	92 (92%)	46 (73%)	38 (73.1%)	21 (65.6%)	64 (74.4%)	16 (76.2%)	18 (46.2%)
BMI (mean ± SD)	27 ± 5	28 ± 5	28 ± 4	25 ± 4	25 ± 4	27 ± 6	25 ± 3	26 ± 5
30-day mortality (%)	26 (7%)	1 (1%)	5 (7.9%)	3 (5.8%)	3 (9.4%)	6 (7%)	7 (33%)	1 (2.6%)
Length of ICU stay (days, median ± IQR)	3 (1–5)	2 (1–3)	2 (1–4)	3 (1–4)	3 (2–5)	3 (2–6)	9 (6–10)	2 (1–4)
Days until extubation (median ± IQR)	0 (0–1)	0 (0–1)	0 (0–1)	0 (0–1)	0 (0–1)	0 (0–1)	3 (1–8)	0 (0–1)
EuroScore (mean ± SD)	6 ± 4	5 ± 3	6 ± 4	4 ± 4	8 ± 4	7 ± 5	12 ± 3	6 ± 5
7-day Follow-Up After ICU Discharge (%)	192 (49%)	47 (47%)	32 (51%)	27 (52%)	14 (44%)	50 (58%)	11 (52%)	11 (28%)
**Surgical characteristics**								
Cardiopulmonary Bypass Time, min (median ± IQR)	135 (105–165)	105 (75–135)	135 (105–165)	135 (105–210)	150 (135–210)	165 (135–210)	105 (105–158)	75 (75–135)
Deep Hypothemic Cardiac Arrest (%)	47 (12%)	0	1 (2%)	0	0	45 (52%)	0	1 (3%)
Selective Antegrade Cerebral Perfusion (%)	45 (11%)	0	0	0	0	43 (50%)	0	0
Intraoperative CPR (%)	5 (1%)	0	2 (3%)	1 (2%)	0	2 (2%)	0	0
Postoperative ECMO (%)	15 (4%)	2 (2%)	3 (5%)	5 (10%)	1 (3%)	3 (4%)	0	1 (3%)
Postoperative IABP (%)	0	0	0	0	0	0	0	0
Postoperative Impella (%)	1 (0.2%)	0	0	1 (2%)	0	0	0	0
Postoperative RVAD (%)	4 (1%)	0	0	0	0	0	4 (19%)	0
**Complications**								
Sepsis (%)	8 (2%)	0	2 (3%)	1 (2%)	2 (6%)	1 (1%)	1 (5%)	1 (3%)
ARDS (%)	2 (1%)	0	1 (2%)	1 (2%)	0	0	0	0
AKI, overall (%)	158 (40%)	33 (33%)	21 (33%)	20 (39%)	17 (53%)	37 (43%)	17 (81%)	13 (33%)
*KDIGO I (%)*	129 (33%)	29 (29%)	18 (29%)	16 (31%)	16 (50%)	24 (28%)	14 (67%)	12 (31%)
*KDIGO II (%)*	19 (5%)	3 (3%)	1 (2%)	3 (6%)	1 (3%)	10 (12%)	1 (5%)	0
*KDIGO III (%)*	10 (3%)	1 (1%)	2 (3%)	1 (2%)	0	3 (3%)	2 (10%)	1 (3%)
*RRT (%)*	14 (4%)	2 (2%)	1 (2%)	0	2 (6%)	4 (5%)	3 (14%)	2 (5%)
Intraoperative fluid balance (mL, mean ± SD)	3234 ± 1074	2761 ± 1007	3225 ± 1321	3253 ± 926	3059 ± 1231	3331 ± 1319	4365 ± 1502	3100 ± 1666
Weight gain at the end of surgery (kg, mean ± SD)	4.7 ± 1.8	3.6 ± 1.9	4.1 ± 1.3	4.2 ± 1.5	5.1 ± 3.7	4.7 ± 2	5.2 ± 3.1	3.6 ± 2.8
ALF (%)	3 (1%)	0	1 (2%)	1 (2%)	1 (3%)	0	0	0
Low-Cardiac Output Syndrome (%)	43 (11%)	6 (6%)	5 (8%)	7 (14%)	5 (15%)	6 (7%)	12 (57%)	2 (6%)
Reoperation within first 7 days (%)	53 (14%)	8 (8%)	8 (13%)	4 (8%)	3 (9%)	18 (21%)	9 (43%)	3 (8%)

*AV* Aortic valve, *AKI* acute kidney injury, *ALF* acute liver failure, *ARDS* acute respiratory distress syndrome, *BMI* body mass index, *CABG* Coronary artery bypass grafting, *CPR* cardiopulmonary resuscitation, *ECMO* extracorporeal membrane oxygenation, *IABP* intra-aortic balloon pump, *ICU* intensive care unit, *KDIGO* Kidney Disease: Improving Global Outcomes, *kg* kilogram, *LVAD* Left ventricular assist device; *MV* Mitral valve, *RRT* Renal replacement therapy; *RVAD* Right ventricular assist device

**Fig. 2:**
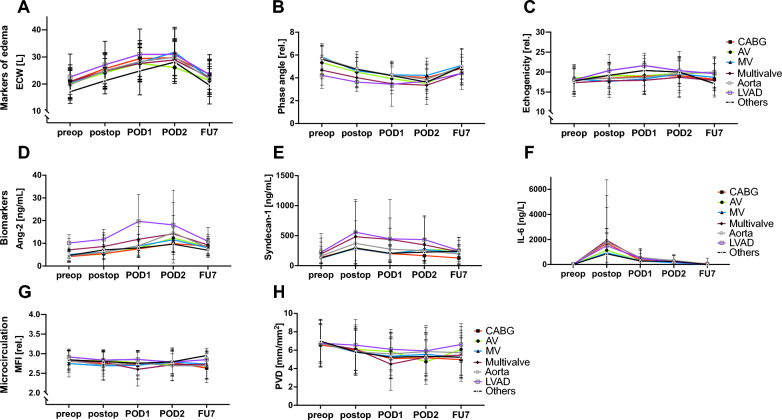
Time course of study variables, grouped by surgical type, on edema, serum biomarkers, and sublingual microcirculation: **A** Extracellular water obtained from body impedance electrical analysis, **B** Phase angle obtained from BIA, **C** Echogenicity obtained from tissue ultrasound, **D** Angiopoietin-2 in serum, **E** Syndecan-1 in serum, **F** Interleukin-6 in serum, **G** Microvascular flow index obtained from sublingual microcirculation assessment, **H** Perfused vessel density obtained from sublingual microcirculation assessment (POD: postoperative day, FU7 = follow-up on day 7 post ICU discharge)

ECW increased in all patient groups from (20 ± 6L) preoperatively to (29 ± 7L; *P* < 0.001; see Fig. 2) postoperative day (POD) 2. At the 7-day follow-up (7FU) measurement, all patient groups showed a reduction in ECW back to baseline (22 ± 6L). Phase angle dropped from 5.6 ± 2.5AU preoperatively to 3.8 ± 1.2AU on POD2, while returning to baseline levels at FU7 (5.1 ± 3.3). The echogenicity derived from ultrasound measurement of subcutaneous tissue did not alter over time; except for patients undergoing LVAD placement who had a modest increase in echogenicity from 18 ± 3AU preoperatively to 22 ± 8AU on POD1. Angiopoietin-2 (Ang-2) increased in all patients from 5.1 ± 4.4 ng/mL at baseline to a maximum of 12.8 ± 9.7 ng/mL on POD2, while the LVAD patients showed the most prominent increase. On FU7, Ang-2 levels were at baseline levels (8.5 ± 4.2 ng/mL). Serum syndecan-1, a marker for glycocalyx shedding, showed the highest levels in LVAD (224 ± 170 preop to 557 ± 544 ng/mL postop) and multivalve (196 ± 269 preop to 482 ± 587 ng/mL postop) patients. Interleukin-6 (IL-6) significantly increased in all surgical groups postoperatively (12 ± 52 at baseline to 1449 ± 3366 ng/L), with a reduction back to preoperative levels on POD1. No significant changes in microvascular flow index (MFI) and perfused vessel density (PVD) occurred.

**Fig. 3:**
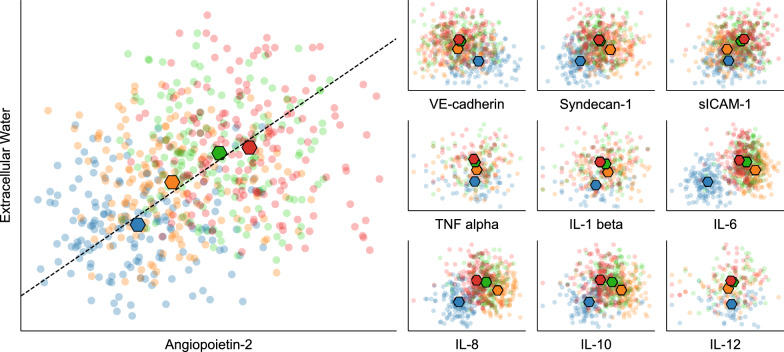
Correlation between biomarkers and extracellular water preoperatively (blue), postoperatively (orange), on POD1 (green) and POD2 (red) with the oversized diamonds showing the average for the respective time of measurement. The respective biomarker is represented on the x-axis and extracellular water is represented on the y-axis

Correlation of endothelial and inflammatory biomarkers showed a positive correlation of Ang-2 with ECW (correlation based on averaged time points of daily measurements: r = 0.959, *P* = 0.041; unsystematic correlation across all patients and without accounting for time of measurement: r*r*= 0.32; *P* < 0.001). No meaningful correlations were found for the other biomarkers sVE-Cadherin, syndecan-1, sICAM-1, TNF-α, IL-1β, IL-6, IL-8, IL-10, and IL-12 (see Fig. 3).

Univariate analysis based on the classification on Ang-2 levels (according to the 75th percentile) showed that patients with high Ang-2 had a significantly increased ECW postoperatively until POD3 (30.4 ± 8.1 vs. 27.4 ± 6.6L on POD1; *P* < 0.001; see Table 2), while phase angle was lower in high Ang-2 patients postoperatively and on POD1 (POD1 3.6 ± 1.2 vs. 4.2 ± 1AU; *P* < 0.001). Echogenicity of subcutaneous tissue was elevated in high Ang-2 patients on POD2 and POD3 (POD2: 21 ± 5.6 vs. 19 ± 3.9AU; *P* < 0.001). IL-6 was increased in high Ang-2 patients postoperatively and on POD1 (postop 2405 ± 4673 vs. 1127 ± 2727 ng/L; *P* = 0.001), while IL-8 was only increased on POD1. C-reactive protein (CRP) was higher postoperatively until POD2 (POD1: 91 ± 49 vs. 73 ± 32 mg/dL; *P* = 0.001). No significant differences were detected in microcirculatory variables. The endothelial biomarkers sVE-Cadherin, syndecan-1, and sICAM-1 were increased in high Ang-2 patients throughout the time points of measurement (POD1: sVE-Cadherin 2.33 ± 0.49 vs. 2.09 ± 0.39 ng/mL; syndecan-1 355 ± 485 vs. 208 ± 157 ng/mL; sICAM-1 342 ± 103 vs. 289 ± 158 ng/mL; all *P* < 0.01). Lactate was higher in the high Ang-2 group postoperatively to POD3 (POD1: 2.8 ± 2.4 vs. 2 ± 1.7 mmol/L; *P* < 0.001). SAPS-II and SOFA scores were increased in the high Ang-2 group, while fluid in- and output did not show any differences postoperatively to POD3.

**Table 2:** Markers of edema, inflammatory and endothelial biomarkers, microcirculation variables, ICU scores, and data on fluid homeostasis postoperatively based on the classification in high Ang-2 and low Ang-2 patients

	Low Ang-2 (< 10.9 ng/mL)	High Ang-2 (≥ 10.9 ng/mL)	*P*-value
Markers of Edema			
ECW [L]			
*Postop*	24 ± 5.6	26.6 ± 7.1	***P* < 0.001**
*POD1*	27.4 ± 6.6	30.4 ± 8.1	***P* < 0.001**
*POD2*	28.7 ± 6.5	31 ± 8.4	***P* = 0.033**
*POD3*	28 ± 7	31.9 ± 9.2	***P* = 0.005**
Phase angle [AU]			
*Postop*	4.7 ± 1.2	4 ± 1	***P* < 0.001**
*POD1*	4.2 ± 1	3.6 ± 1.2	***P* < 0.001**
*POD2*	3.9 ± 1.1	3.7 ± 1.4	*P* = 0.398
*POD3*	3.6 ± 1	3.5 ± 2	*P* = 0.784
Echogenicity [AU]			
*Postop*	18.7 ± 3.6	18.8 ± 3.8	*P* = 0.348
*POD1*	18.7 ± 3.7	19.3 ± 3.8	*P* = 0.115
*POD2*	19 ± 3.9	21 ± 5.6	***P* = 0.002**
*POD3*	19.3 ± 3.9	20.9 ± 5.6	***P* = 0.044**
Markers of Inflammation			
Interleukin-6 [ng/L]			
*Postop*	1127 ± 2727	2405 ± 4673	***P* = 0.001**
*POD1*	316 ± 467	628 ± 739	***P* < 0.001**
*POD2*	278 ± 973	336 ± 519	*P* = 0.655
*POD3*	201 ± 1041	1555 ± 8734	*P* = 0.131
Interleukin-8 [ng/L]			
*Postop*	279 ± 1675	368 ± 473	*P* = 0.6
*POD1*	61 ± 65	195 ± 304	***P* < 0.001**
*POD2*	69 ± 317	126 ± 260	*P* = 0.199
*POD3*	42 ± 55	341 ± 1584	*P* = 0.063
C-reactive Protein [mg/dL]			
*Postop*	21 ± 24	60 ± 82	***P* = 0.032**
*POD1*	73 ± 32	91 ± 49	***P* = 0.001**
*POD2*	189 ± 64	217 ± 70	***P* = 0.004**
*POD3*	209 ± 80	232 ± 79	*P* = 0.084
Microcirculation			
Microvascular Flow Index (MFI)			
*Postop*	2.8 ± 0.3	2.7 ± 0.4	*P* = 0.071
*POD1*	2.7 ± 0.3	2.8 ± 0.3	*P* = 0.673
*POD2*	2.7 ± 0.3	2.8 ± 0.3	*P* = 0.424
*POD3*	2.8 ± 0.3	2.9 ± 0.3	*P* = 0.146
Perfused Vessel Density (PVD, mm/mm^2^)			
*Postop*	5.9 ± 2.4	6.3 ± 2.8	*P* = 0.152
*POD1*	5.3 ± 2.3	5.7 ± 2.4	*P* = 0.12
*POD2*	5.4 ± 2.6	5.9 ± 2.5	*P* = 0.242
*POD3*	5.9 ± 2.4	6.2 ± 2.2	*P* = 0.457
Biomarkers			
sVE-Cadherin [ng/mL]			
*Postop*	2.08 ± 0.44	2.28 ± 0.58	***P* < 0.001**
*POD1*	2.09 ± 0.39	2.33 ± 0.49	***P* < 0.001**
*POD2*	2.07 ± 0.37	2.3 ± 0.42	***P* < 0.001**
*POD3*	2.05 ± 0.35	2.22 ± 0.46	**P = 0.008**
Syndecan-1 [ng/mL]			
*Postop*	307 ± 314	433 ± 428	***P* = 0.002**
*POD1*	208 ± 157	355 ± 485	***P* < 0.001**
*POD2*	214 ± 158	406 ± 465	***P* < 0.001**
*POD3*	226 ± 170	597 ± 819	***P* < 0.001**
sICAM-1 [ng/mL]			
*Postop*	234 ± 97	297 ± 311	***P* = 0.002**
*POD1*	289 ± 158	342 ± 103	***P* = 0.002**
*POD2*	317 ± 110	393 ± 119	***P* < 0.001**
*POD3*	344 ± 119	439 ± 178	***P* < 0.001**
Lactate [mmol/L]			
*Postop*	3.3 ± 2.6	4.8 ± 2.9	***P* < 0.001**
*POD1*	2 ± 1.7	2.8 ± 2.4	***P* < 0.001**
*POD2*	1.3 ± 1	1.6 ± 1.1	***P* = 0.015**
*POD3*	1.1 ± 0.5	1.6 ± 1.7	**P = 0.003**
ICU Scores			
SAPS-II			
*Postop*	38 ± 15	45 ± 14	***P* < 0.001**
*POD1*	21 ± 15	34 ± 19	***P* < 0.001**
*POD2*	22 ± 14	35 ± 19	***P* < 0.001**
*POD3*	22 ± 14	37 ± 18	***P* < 0.001**
SOFA			
*Postop*	9 ± 3	10 ± 3	***P* < 0.001**
*POD1*	7 ± 3	9 ± 3	***P* < 0.001**
*POD2*	7 ± 3	9 ± 3	***P* < 0.001**
*POD3*	7 ± 3	10 ± 3	***P* < 0.001**
Fluids			
Fluid input [mL]			
*POD1*	1075 ± 772	1054 ± 698	*P* = 0.815
*POD2*	888 ± 653	923 ± 1100	*P* = 0.745
*POD3*	834 ± 595	740 ± 482	*P* = 0.273
Output [mL]			
* POD1*	607 ± 504	718 ± 657	*P* = 0.078
* POD2*	572 ± 507	696 ± 686	*P* = 0.103
* POD3*	741 ± 591	705 ± 619	*P* = 0.698

Bold indicates statistically significant results

*AU* arbitrary units, *ECW* Extracellular water, *POD* postoperative day, *SAPS-II* Simplified Acute Physiology Score-II; *SOFA* Sequential organ failure assessment score, *SAPS* Simplified Acute Physiology Score

We found a statistically significant association between Ang-2 and ECW, both in the unadjusted and adjusted statistical models (see Table 3). After controlling for common confounders of edema formation in cardiac surgery patients, a coefficient of 0.24 (95% CI 0.163, 0.317; *P* < 0.001) for Ang-2 was calculated using a GEE model which takes into account the dynamics of patient measurements on multiple days (an increase in Ang-2 of 1 ng/mL was, therefore, associated on average, with a 0.24 L increase in ECW). Moreover, aortic valve, multivalve, and “other” surgeries showed a negative coefficient in their association with ECW as compared to the reference category of coronary artery bypass grafting. CPB time had a positive coefficient of 0.033 (95% CI 0.02, 0.047; *P* < 0.001), while the perioperative transfusion of PRBCs and normal LVEF variables showed negative coefficients.

**Table 3:** Univariable and multivariable models to examine the association between angiopoietin-2 (Ang-2) and extracellular water (ECW) using generalized estimating equations (GEE); the multivariable model was adjusted for chronic kidney disease, normal left ventricular ejection fraction, type of surgical procedure, duration of cardiopulmonary bypass (CPB), diuretic use, and packed red blood cell (PRBC) transfusion

	Unadjusted coefficient (95% CI)	*P*-value	Adjusted coefficient (95% CI)	*P*-value
Angiopoietin-2	0.242 (0.165, 0.319)	***P* < 0.001**	0.24 (0.163, 0.317)	***P* < 0.001**
Surgery type				
Aortic valve^#^			− 1.720 (− 3.314, − 0.125)	***P* = 0.034**
Mitral valve^#^			− 1.817 (− 3.821, 0.186)	*P* = 0.075
Multivalve^#^			− 3.223 (− 5.312, − 1.134)	***P* = 0.002**
Aortic surgery^#^			− 1.644 (− 3.416, 0.129)	*P* = 0.069
Left ventricular assist devices^#^			0.382 (− 3.281, 4.045)	*P* = 0.838
Others^#^			− 4.037 (− 6.117, − 1.957)	***P* < 0.001**
Cardiopulmonary bypass time			0.033 (0.020, 0.047)	***P* < 0.001**
PRBC Transfusion			− 2.348 (− 3.711, − 0.985)	***P* = 0.001**
Normal LVEF			− 2.205 (− 3.385, − 1.025)	***P* < 0.001**
Diastolic dysfunction			0.033 (− 1.048, 1.518)	*P* = 0.719
Chronic kidney disease			− 1.230 (− 2.708, 0.249)	*P* = 0.103
Diuretic use			− 0.048 (− 1.209, 1.113)	*P* = 0.935

Bold indicates statistically significant results

^#^vs. coronary artery bypass grafting

In an unadjusted model, mortality was increased in the high Ang-2 group compared to low Ang-2 patients (HR 4.031 [95% CI 1.556, 10.44], *P* < 0.001). However, the number of absolute events limited precise multivariable modelling to support definitive conclusions regarding mortality.

**Fig. 4:**
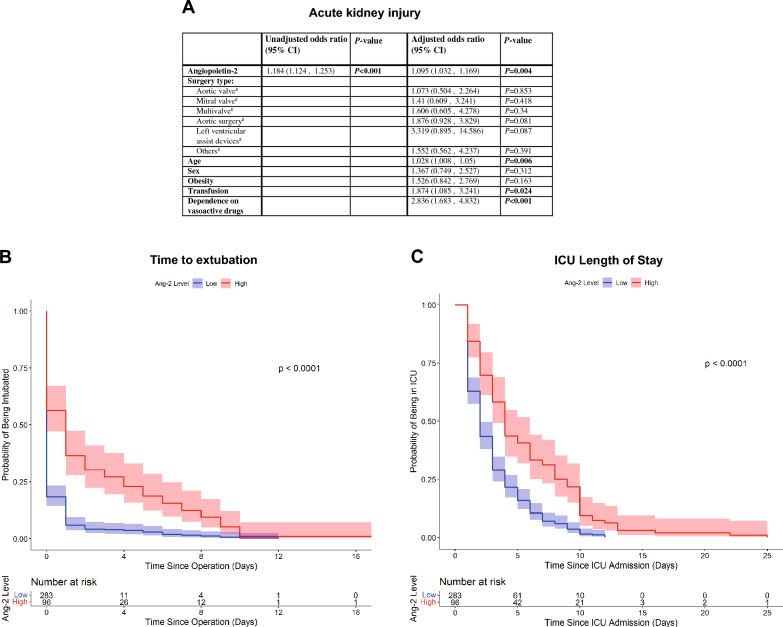
Secondary analyses. **A** Uni- and multivariable logistic regression models for the association of Ang-2 and postoperative, acute kidney injury (AKI). In the multivariable model, common risk factors associated with AKI after cardiac surgery were controlled for (^#^vs. coronary artery bypass grafting). **B** Using Kaplan–Meier curves, time to extubation was significantly prolonged in patients showing high serum Ang-2 levels (*P* < 0.0001). **C** Discharge from the ICU occurred later in patients with high Ang-2 levels (*P* < 0.0001)

AKI was studied as a secondary outcome (see Fig. 4A). After adjusting for common risk factors of AKI in cardiac surgery, an increase of 1 ng/mL in Ang-2 was associated with a 9.5% (95% CI 1.032, 1.169; *P* = 0.004) increase in the odds of developing perioperative AKI. While there was no association between type of surgery and AKI, age, transfusion of PRBCs, and dependence on vasoactive medication were significantly associated with increased odds of AKI.

Higher Ang-2 was associated with a delayed time to extubation both in the Kaplan–Meier analysis and the Cox proportional hazards models (see Fig. 4B and Additional file 4: Table S3). On POD4, 26 patients remained intubated in the high Ang-2 group vs. 11 patients with low Ang-2 levels. Considering Ang-2 as a continuous variable and controlling for risk factors of delayed extubation after cardiac surgery resulted in a HR of 0.939 (95% CI 0.92, 0.96; *P* < 0.001); consequently, an increase of 1 ng/mL of Ang-2 was associated with a 6.1% decrease in the hazard of being extubated. Age, CPB time, and postoperative hyperglycemia were also significantly associated with decreased hazard of extubation.

Kaplan–Meier analysis showed a longer ICU LOS in the high Ang-2 patients (*P* < 0.001), while an unadjusted Cox model produced a HR of 0.928 (95% CI 0.907, 0.949; *P* < 0.001; see Fig. 4C and Additional file 5: Table S4, Additional file 6: Table S5). In the multivariable Cox model, only LVAD placement, a history of PRBC transfusion, and continued invasive ventilation strategies were significantly associated with decreased hazard of ICU discharge, while an improved P-F-ratio was associated with increased hazard of discharge.

After adjusting for risk factors, an increase of 1 ng/mL in Ang-2 was associated with a 20.8% increase in the odds (95% CI 1.126, 1.306; *P* < 0.001) of continuous postoperative dependence on vasoactive drugs beyond POD 1 (see Additional file 6: Table S5). Other factors associated with increased odds of dependence on pharmacological hemodynamic support were age, RV dysfunction, and a longer CPB time, while a normal LVEF was associated with decreased odds.

The association between Ang-2 and P-F ratio as a marker for oxygenation was only statistically significant in an unadjusted model (*P* = 0.047), while adjusting for confounders using GEE did not support this finding (*P* = 0.081; see Additional file 7: Table S6).

**Fig. 5:**
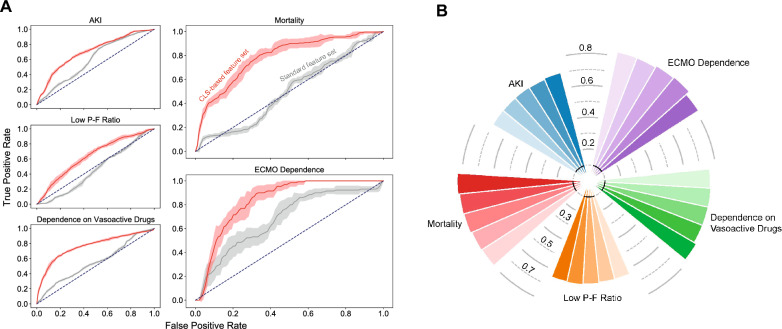
Machine learning models predicting complications based on common risk factors, and by adding a combination of Ang-2 and body impedance electrical analysis-derived measurements to the risk factors. **A** shows ROC-AUC of machine learning (ML) algorithms to predict acute kidney injury (AKI), low oxygenation index (P/F-ratio), dependence on vasoactive drugs, mortality, and postoperative dependence on ECMO from standard risk factors (grey curve). The red curves represent an augmented feature set, adding the phenotype of capillary leak to the standard risk factors (with Ang-2 and body impedance electrical analysis-derived measurements), thus showing a significant improvement in predicting the respective postoperative complication (blue dotted lines: random selection). **B** shows different cross-validation methods and their respective ROC-AUC to predict complications derived from ML. Models could maintain their robust performance in terms of ROC-AUC regardless of the splitting approaches used in various cross-validation strategies. Color from dark to light (counter-clockwise) in each segment: repeated train-test splitting validation, standard cross-validation, stratified cross-validation, leave-one-patient-out cross-validation, and leave-one-surgery-out cross-validation

ML models based on general risk factors showed an AUC of 0.6 for AKI, 0.46 for low P-F-ration, 0.55 for dependence on vasoactive substances, 0.5 for mortality, and 0.64 for postoperative dependence on ECMO. Combining these risk factors with Ang-2 and body impedance electrical analysis-derived edema measurements led to a significant improvement in prediction of the respective complication (all *P* < 0.001; see Fig. 5A and Additional file 8: Fig. S1). Dependence on ECMO then showed an AUC of 0.84, mortality 0.82, dependence on vasoactive medication 0.77, AKI 0.7, and low P-F-ratio 0.65. Comparing the different cross-validation strategies, standard cross-validation showed the best AUC to predict the complications except for AKI model which had its best AUC in a stratified cross-validation approach (see Fig. 5B).

## Discussion

The results from our prospective, observational study can be summarized as follows: A) We were able to provide longitudinal data on the development and course of inflammatory edema formation after cardiac surgery. B) By studying biomarkers associated with the pathophysiology of capillary leak, Ang-2 best correlated with endothelial injury, inflammation, and glycocalyx shedding. However, we did not identify an association of Ang-2 with sublingual microcirculation. C) An increase in Ang-2 was linked to cardiovascular, pulmonary, and renal complications after cardiac surgery, directing further attention to the deleterious effects of capillary leak. D) Machine learning algorithms for postsurgical complications were augmented in its predictive potential with ECW and Ang-2, stressing the relevance of capillary leak. Diagnostic capabilities were improved beyond classic risk factors and early patient identification with targeted interventions can be envisioned.

Mechanisms related to CLS have been investigated in cell and animal models. While an inflammatory phenotype increases vascular permeability [18], the integrity of inter-endothelial adhesions become compromised [19]. A decreased intracellular cAMP levels lead to reduced inter-cellular interaction and vascular barrier function [18]. By targeting endothelial cAMP, experimental pharmacological mechanisms (e.g., phosphodiesterase-IV inhibition) have been successfully tested to attenuate inflammation-triggered fluid extravasation in pre-clinical models [20]. In a clinical study examining seven critically ill patients, Flemming et al. demonstrated that components of endothelial cell junctions (sVE-Cadherin) are detectable in the patients’ serum and its concentration decreased with resolution of disease [21]. In cardiac surgery, mechanical injury and shear stress from extracorporeal blood flow may further exacerbate vascular injury together with the systemic inflammatory response to CPB. Rehm et al. were able to demonstrate in 18 patients undergoing surgery on CPB that endothelial glycocalyx is shedded while serum markers like heparan sulfate and syndecan-1 markedly increased [22]. Among mediators of vascular permeability, Ang-2 has recently attracted attention. It can be released from Weibel-Palade bodies from the endothelium while its binding to the Tie-2 receptor may increase the endothelial barrier’s response to pro-inflammatory cytokines [23]. By binding of Ang-2 to the Tie-2 receptor, the barrier-protective effects of Ang-1, targeting the same receptor, are abrogated [24, 25]. In their study on 25 patients undergoing surgery on CPB, Clajus et al. showed that Ang-2 levels increased from pre- to postoperatively. Ang-2 correlated with signs of fluid overload and CPB time. Furthermore, serum from 25 patients with high Ang-2 levels disrupted endothelial cell layers in-vitro [26]. In a recent study investigating a heterogenous cohort of 200 critically ill patients, we were able to analyze multiple biomarkers reflective of capillary leak. We found that Ang-2, ICAM-1, sVE-Cadherin, and syndecan-1 were significantly elevated in patients with phenotypical CLS, while Ang-2 showed the best characteristics for being included in a clinical scoring system[4]. With our current study, we were able to expand the knowledge on CLS and Ang-2 in the field of cardiac surgery. Importantly, we not only found an association of Ang-2 with ECW—the gold standard assessment of edema formation and capillary leak—but we also identified a link of Ang-2 with relevant complications, common in patients presenting with fluid overload. Of note, we used BIA as a non-invasive tool to assess the fluid compartments, increasing the relevance of our findings. Previously, BIA was compared against invasive measurements of ECW [27], while it was furthermore suggested to be useful in the ICU setting aiding decision beyond fluid status (e.g., nutritional support, drug dosing) [28].

Previous studies in general ICU cohorts have linked Ang-2 with acute lung injury and multi-organ dysfunction [29, 30]. In a study of 21 post-CPB patients, the increase in postoperative Ang-2 was higher in patients who developed AKI [31] and an altered Ang-2/Ang-1 ratio was correlated with higher odds for prolonged mechanical ventilation [10]. In 30 neonates less than two weeks of age undergoing congenital cardiac repair, patients with a greater rise in Ang-2 showed a higher chance for a composite of adverse outcomes after cardiac surgery [32]. Secondary analyses from our cohort further highlight the predictive relevance of CLS for hemodynamic, renal, and pulmonary complications. Here, AKI, the duration of mechanical ventilation, the need for pharmacologic hemodynamic support, and the LOS in the ICU were affected. Especially AKI presents a considerable challenge in cardiac surgery and was previously shown to increase perioperative morbidity and mortality, with only limited evidence-based strategies to reduce its risk [33]. The AUC of 0.6 in the ROC analysis from the ML models based on standard risk factors stresses the difficulty to accurately diagnose AKI, while adding CLS in our prediction model significantly improved its robustness. With our data we are thus able to demonstrate clinical relevance of CLS for complications after cardiac surgery.

Contrary to our hypothesis we found no relevant association of Ang-2 with markers of sublingual microcirculation. Previously, cardiac surgery as well as mechanical circulatory support have been shown to lead to microcirculatory alterations [34–37]. In a study by Wadowski et al., a rarefication of the capillary density was observed in LVAD patients which was also associated with bleeding complications [40]. Dekker et al. demonstrated that increased Ang-2 levels correlated moderately with indices of reduced sublingual microcirculatory perfusion in 17 patients after surgery on CPB [38]. In our prospective cohort of 405 patients, there was no meaningful association of Ang-2 with metrics of sublingual microcirculation. Visualization of the microvascular network with handheld high-resolution cameras, as used in our study, has enabled direct insight into the complex network of microvascular perfusion. Important to note, microcirculation is a conserved, yet locally auto-regulated system by various mediators. Therefore, heterogeneity is not unexpected, thereby presenting a challenge to interpret findings from the sublingual region and correlate it to the organ state. Of note, microcirculatory alterations may occur as early as on or immediately after CPB [39]. However, in our study we focused on postoperative measurements in the ICU, and thus may have missed intraoperative alterations. However, as we found differences in lactate levels in our patients, either some microcirculatory heterogeneity or a degree of impaired lactate kinetics may have been present.

We employed various statistical techniques to account for potential confounders. The advantage of our dataset is that it was curated longitudinally with up to 31 consecutive measurements for individual patients in the ICU. By employing GEE, we were able to account for the edema dynamics, enabling us to reference it to respective changes in Ang-2. Hence, conclusions from multiple timepoints measured in the same patients are possible. Moreover, controlling for common risk factors for edema formation after cardiac surgery (like a reduced LVEF, CKD, or diastolic dysfunction) did not alter the association of Ang-2 and ECW. To account for confounders in our secondary analyses, GEE, Cox proportional hazard, and logistic regression models were used. We were able to identify statistically significant associations for Ang-2 with the development of AKI, dependence on vasoactive drugs, as well as a prolonged time to extubation (with a trend to reduced oxygenation index and length of stay in the ICU related to high Ang-2). Overall mortality was not different in our adjusted models despite a trend in univariate analyses. To further boost the predictive capabilities, we complemented our multivariable statistical approach with methods of machine learning. The results show that common risk factors failed to accurately predict complications after cardiac surgery. By adding CLS to the risk assessment, the predictive capabilities for the respective complications were improved (e.g., ROC-AUC for AKI from 0.6 to 0.7), thus enabling a more robust classification system. Our results clearly stress the importance to diagnose CLS and demonstrate its relevance for complications. In general, ML models can help to predict the individual patient course, and its utility may be further envisioned by automatic data computation from EMR. Although the ML model is mathematically more complex than the applied statistics, it is qualitatively different by allowing for predictions that have not been considered during model training. Thereby, the algorithm prototypes a novel system that could inform future automated clinical decision making and to triage patients. Of note, ML models have previously been published to predict the need for ECMO dependence after cardiac surgery [41]. In particular, some of these models merely rely on patients’ EMR data, and thus not requiring additional input from BIA or serum measurements. In our study, we were able to further refine the performance of our models with additional input from BIA, highlighting the importance of the fluid homeostasis for the patient’s prognosis. Importantly, the efforts needed for BIA may be comparable to an electrocardiogram with non-invasive application of electrodes to the patients’ skin to perform short measurements while the utility of this technique may reach beyond mere volume status assessment [12].

As an important differentiation to capillary leak, we postulate that vasoplegic syndrome should be viewed as a different entity. The pathomechanism of vasoplegia after cardiac surgery is thought to be related to an inflammation-related vasodilation, leading to a decrease in systemic vascular resistance. This state should be treated with vasoactive medication instead of fluid administration [42]. In contrast, CLS refers to a microvascular injury leading to leaky blood vessels, resulting in loss of fluid to third spaces. A reduction in venous return seen in vasoplegia may mimic the picture of fluid loss by pooling blood volume in intravascular, yet recruitable spaces. Of note, in our study we used advanced hemodynamic monitoring to differentiate states of low systemic vascular resistance, reduced contractility, and hypovolemia to allow for targeted treatment. The fact that an increase in Ang-2 was also associated with a higher chance of vasopressor requirement in our study may be the result of systemic inflammation which could have triggered not only the vascular leak but also caused vasodilatation in these patients. Interestingly, a recent study by Joffre et al. indicated that commonly used catecholaminergic drugs used to treat vasoplegia may also reduce inflammatory endothelial permeability in-vitro [43] in its cAMP-dependent downstream signaling comparable to experimental approaches used for CLS [44]. Despite marked differences, the complex interaction of the two pathological states deserves further attention.

The strength of our study is that we were able to follow our patients throughout their hospital course. We obtained pre- and postoperative, daily ICU, and a 7-day follow-up measurement in a large cohort. We were able to draw conclusions on the dynamics of fluid extravasation and its resolution throughout the patients’ hospital stay. Furthermore, we employed multiple techniques of non-invasive assessment related to microvascular physiology as well as extensive statistical modelling. The reported rate of complications (e.g., a 40% rate of AKI) in our patients depicts a sick, yet typical cardiac surgery cohort. Certain limitations should be considered when interpreting our data. First, vascular permeability presents a continuum of health and disease. A certain, yet often unknown degree of permeability is essential to maintain homeostasis, enabling exchange processes across the vascular barrier. However, it remains challenging to determine an individual cut-off for permeability to be considered a pathological state. Second, vascular permeability is typically assessed by surrogate markers. We are not aware of an easy, non-invasive, reliable and well-established visualization of fluid extravasation in patients. Therefore, we included multiple metrics of vascular permeability and edema formation, comprising ECW, the phase angle as a means for membrane integrity, ultrasound measurements, sublingual microscopy as well as inflammatory and endothelial biomarkers. The studied biomarkers confirmed the etiology CLS with its hallmarks of glycocalyx shedding (i.e., increase in serum syndecan-1), inflammation (i.e., IL-6), mediators of vascular permeability (i.e., Ang-2), as well as an increase in cleaved inter-endothelial junction proteins in the serum (i.e., sVE-Cadherin, ICAM-1).

## Conclusions

With our study, we can draw conclusions from the relationship of biomarkers, edema formation, and complications after cardiac surgery. Our results suggest a strong relationship of Ang-2 with perioperative CLS and show a robust link to postoperative complications like AKI. With the expanding knowledge on CLS, interventional studies may be conceptualized for targeted interventions in the Ang-2 axis, thereby intending to minimize complications after cardiac surgery.

### Supplementary Information

**Additional file 1. **Additional methods.

**Additional file 2:** **Table S1.** Past medical history and medications.

**Additional file 3:** **Table S2.** Perioperative echocardiographic metrics.

**Additional file 4:** **Table S3.** Uni- and multivariable Cox proportional hazards models for time to extubation. Common risk factors were controlled for in the multivariable model.

**Additional file 5:** **Table S4.** Uni- and multivariable models for ICU LOS. A Cox proportional hazards model was used to control for common risk factors for delayed discharge from ICU level of care.

**Additional file 6:** **Table S5.** Uni- and multivariable logistic regression models for postoperative dependence on vasoactive drugs. The multivariable model was used to control for risk factors for postoperative dependence on vasoactive drugs.

**Additional file 7:** **Table S6.** Uni- and multivariable models for the association of Ang-2 and the P-F-ratio. Generalized estimating equations (GEE) were used to model this association by accounting for repeated measurements on patients. Established confounders were controlled for. In case, patients were not treated invasively, F_i_O_2_ was estimated according to the estimated deliverable fraction of oxygen using non-invasive devices.

**Additional file 8:** **Fig S1. **Performances of different ML models showing the basic feature set (only containing accepted risk factors for the respective complication) in grey with its comparison to an augmented feature set in red, taking the diagnosis capillary leak into account. Significant improvements of all metrics were observed on models trained using the augmented feature sets.

**Additional file 9: Fig. S2. a** Standard cross validation, **b** leave-one-patient-out cross validation, **c** leave-one-surgery out cross validation.

## Data Availability

The datasets used and/or analyzed during the current study are available from the corresponding author on reasonable request.

## Abbreviations

ACEi: Angiotensin converting enzyme inhibitors
AKI: Acute kidney injury
ALF: Acute liver failure
Ang-2: Angiopoietin-2
ARB: AT_1_-receptor blockers
AU: Arbitrary units
AUC: Area under the curve
AV: Aortic valve
CABG: Coronary artery bypass grafting
CLS: Capillary Leak Syndrome
CKD: Chronic kidney disease
CPB: Cardiopulmonary bypass
DHCA: Deep hypothermic cardiac arrest
ECMO: Extracorporeal membrane oxygenation
ECW: Extracellular water
ELISA: Enzyme-linked immunosorbent assay
EMR: Electronic medical record
FU7: Follow-up at day seven
GEE: Generalized estimating equations
ICU: Intensive Care Unit
IL: Interleukin
KDIGO: Kidney disease improving global outcomes
LCOS: Low cardiac output syndrome
LOS: Length of stay
LVAD: Left ventricular assist device
LVEF: Left ventricular ejection fraction
ML: Machine Learning
MFI: Microvascular flow index
MV: Mitral valve
NPV: Negative predictive value
OHT: Orthotopic heart transplant
P-F ratio: P_a_O_2_ / F_i_O_2_ ratio
POD: Postoperative day
PPV: Proportion of perfused vessels
PRBC: Packed red blood cells
ROC: Receiver operating characteristics
SOFA: Sequential organ failure assessment
TNF: Tumor necrosis factor
