# Supplementary material for: Angiopoietin-2 is associated with capillary leak and predicts complications after cardiac surgery
Source: Ann Intensive Care. 2023 Aug 8;13:70. doi: 10.1186/s13613-023-01165-2 (PMC10409979; doi:10.1186/s13613-023-01165-2)
Supplement: Supplementary file 1 — Additional file 1. Additional methods. [file 13613_2023_1165_MOESM1_ESM.docx]

**Additional file 1**

**Methods:**

Study measurements:

The collected patient serum was analyzed using ELISA technique (Angiopoietin-2 [Human Angiopoietin-2 Quantikine ELISA kit, #DANG20, R&D Systems, USA]; sVE-Cadherin [VE-Cadherin [Human VE-Cadherin Quantikine ELISA kit, #DCADV0, R&D Systems, USA]; sICAM-1 [Human ICAM-1 Quantikine ELISA kit, #DCD540, R&D Systems, USA]; syndecan-1 [Human Syndecan-1 ELISA kit, #AB46506, Abcam Ltd, United Kingdom]) and FACS (cytokines TNF-α, IL-1ß, IL-6, IL-8, IL-10, and IL-12p70 [Human Inflammatory Cytokine Kit, #551811, BDTM, USA]) according to the manufacturers’ recommendations.

Statistics:

Statistical analyses were performed with SPSS (V26, Chicago, USA), R software (V4.1.2, R Foundation for Statistical Computing, Vienna, Austria), and GraphPad Prism (V9, San Diego, USA). P-values ≤ 0.05 were considered statistically significant. First, univariate analyses were performed. Student’s t-test was used for normally distributed data and Mann Whitney U test for non-normally distributed data. Categorial variables were analyzed with *X^2^* test. High Ang-2 levels were classified according to the 75^th^ percentile. All models were fit using a complete case analysis, and statistical hypotheses were two-sided.

For modelling the association between extracellular water and angiopoietin-2, an unadjusted model was fit followed by a multivariable model adjusting for the following variables: surgery type, normal left ventricular ejection fraction (LVEF), chronic kidney disease, diuretic medications, time of cardiopulmonary bypass, and packed red blood cell (PRBC) transfusion. A generalized estimating equation (GEE) approach was used to account for consecutive measurements on multiple postoperative days. The same GEE approach was used to assess the association between Ang-2 and the secondary outcome of P/F-ratio, adjusting for age, obesity, CPB time, massive PRBC transfusion (defined as more than 10 units), antidiabetic medications, preoperative myocardial infarction, and respiratory tract infection [1].

Logistic regression was used for modelling the relationship of Ang-2 with the secondary outcomes of AKI and continuous dependence on vasoactive medication. The multivariable AKI model was adjusted for age, sex, obesity, surgery type, PRBC transfusion, and dependence on vasoactive drugs [2]. The multivariable model for continuous dependence on vasoactive medication was adjusted for age, normal LVEF, right ventricular dysfunction, CPB time, ACE inhibitor medications, and perioperative atrial fibrillation.

The relationship of Ang-2 and the secondary outcomes of time to ICU discharge and time to extubation were assessed by means of survival analysis. An unadjusted analysis was performed using Kaplan-Meier curves to compare these outcomes between those with high POD1 Ang-2 levels and those with low levels. Unadjusted and adjusted Cox proportional hazards models were fit for Ang-2 as a continuous variable. The multivariable time to ICU discharge model was adjusted for CPB time, postoperative P/F-ratio, surgery type, PRBC transfusion, and prolonged mechanical ventilation[3]. The multivariable time to extubation model was adjusted for age, CPB time, hyperglycemia, and postoperative P/F-ratio [4].

Definitions:

To meet the diagnostic criteria for any severity of diastolic dysfunction, a patient had to have an E/e’ of > 14, a septal e’ velocity < 7 cm/s or lateral e’ velocity <10cm/s, a tricuspid regurgitant jet velocity of > 2.8 m/s, and a left atrial volume index of >34 ml/m^2^. This definition is in accordance with the 2016 Definition of American Heart Association for diastolic dysfunction [5]. Acute kidney injury was classified according the Kidney Disease Improving Global Outcomes (KDIGO) classification.

Clinical Care:

All patients who underwent implantation of a left ventricular assist device (LVAD) received a HeartMate III^TM^ device.

**Literature:**

1. Wang Y, Xue S, Zhu H. Risk factors for postoperative hypoxemia in patients undergoing Stanford A aortic dissection surgery. J Cardiothorac Surg. 2013;8:118.

2. Ramos KA, Dias CB. Acute Kidney Injury after Cardiac Surgery in Patients Without Chronic Kidney Disease. Braz J Cardiovasc Surg. 2018;33:454–61.

3. Zhang X, Zhang W, Lou H, Luo C, Du Q, Meng Y, et al. Risk factors for prolonged intensive care unit stays in patients after cardiac surgery with cardiopulmonary bypass: A retrospective observational study. Int J Nurs Sci. 2021;8:388–93.

4. Suematsu Y, Sato H, Ohtsuka T, Kotsuka Y, Araki S, Takamoto S. Predictive risk factors for delayed extubation in patients undergoing coronary artery bypass grafting. Heart Vessels. 2000;15:214–20.

5. Nagueh SF, Smiseth OA, Appleton CP, Byrd BF, Dokainish H, Edvardsen T, et al. Recommendations for the Evaluation of Left Ventricular Diastolic Function by Echocardiography: An Update from the American Society of Echocardiography and the European Association of Cardiovascular Imaging. J Am Soc Echocardiogr. 2016;29:277–314.
