# Supplementary material for: Angiopoietin-2 is associated with capillary leak and predicts complications after cardiac surgery
Source: Ann Intensive Care. 2023 Aug 8;13:70. doi: 10.1186/s13613-023-01165-2 (PMC10409979; doi:10.1186/s13613-023-01165-2)
Supplement: Supplementary file 2 — Additional file 2: Table S1. Past medical history and medications. [file 13613_2023_1165_MOESM2_ESM.docx]

**Additional file 2: Table S1:**

| **Past Medical History** | *All patients (N=393)* | *CABG (N=100)* | *AV (N=63)* | *MV (N=52)* | *Multivalve (N=32)* | *Aorta (N=86)* | *LVAD (N=21)* | *Others (N=39)* |
| --- | --- | --- | --- | --- | --- | --- | --- | --- |
| Hypertension (%) | 259 (66%) | 78 (78%) | 51 (81%) | 23 (44%) | 21 (66%) | 63 (73%) | 11 (52%) | 12 (31%) |
| Myocardial infarction (%) | 66 (17%) | 42 (42%) | 5 (8%) | 2 (4%) | 3 (9%) | 4 (5%) | 5 (24%) | 5 (12%) |
| Chronic obstructive pulmonary disease (%) | 39 (10%) | 10 (10%) | 6 (10%) | 5 (10%) | 1 (3%) | 13 (15%) | 1 (5%) | 3 (8%) |
| Congestive heart failure (%) | 81 (21%) | 17 (17%) | 15 (23%) | 5 (10%) | 6 (19%) | 10 (12%) | 19 (91%) | 9 (23%) |
| Atrial fibrillation (%) | 104 (27%) | 13 (13%) | 15 (24%) | 19 (37%) | 19 (59%) | 20 (23%) | 10 (48%) | 7 (21%) |
| Endocarditis (%) | 18 (5%) | 0 | 8 (13%) | 4 (8%) | 5 (16%) | 1 (1%) | 0 | 0 |
| Aortic Dissection (%) | 13 (3%) | 0 | 0 | 0 | 0 | 13 (15%) | 0 | 0 |
| Chronic Kidney Disease (%) | 74 (19%) | 18 (18%) | 11 (18%) | 6 (12%) | 11 (34%) | 14 (16%) | 9 (43%) | 5 (13%) |
| Stroke (%) | 49 (13%) | 12 (12%) | 6 (10%) | 2 (4%) | 5 (16%) | 16 (19%) | 2 (10%) | 6 (15%) |
| Pulmonary Hypertension (%) | 74 (19%) | 7 (7%) | 9 (14%) | 8 (15%) | 14 (44%) | 14 (16%) | 12 (57%) | 10 (26%) |
| Non-ischemic Cardiomyopathy (%) | 24 (6%) | 0 | 1 (2%) | 0 | 1 (3%) | 1 (1%) | 16 (76%) | 2 (5%) |
|  |  |  |  |  |  |  |  |  |
| **Medication** | *All patients* | *CABG* | *AV* | *MV* | *Multivalve* | *Aorta* | *LVAD* | *Others* |
| ACE inhibitors / AT_1_ -receptor blockers (%) | 155 (39%) | 52 (52%) | 24 (38%) | 14 (27%) | 12 (38%) | 37 (43%) | 7 (33%) | 9 (23%) |
| ß-Receptor-Blockers (%) | 219 (56%) | 65 (65%) | 37 (59%) | 22 (42%) | 21 (66%) | 47 (55%) | 11 (52%) | 15 (39%) |
| Calcium channel blockers (%) | 88 (22%) | 19 (19%) | 15 (24%) | 4 (8%) | 10 (31%) | 34 (40%) | 2 (10%) | 4 (10%) |
| Diuretics (%) | 155 (39%) | 37 (37%) | 28 (44%) | 20 (39%) | 18 (56%) | 26 (30%) | 16 (76%) | 10 (26%) |
| Statins (%) | 185 (47%) | 78 (78%) | 32 (51%) | 16 (31%) | 12 (38%) | 28 (33%) | 8 (38%) | 11 (28%) |
| Aspirin (%) | 182 (46%) | 84 (84%) | 33 (52%) | 10 (19%) | 9 (28%) | 29 (34%) | 8 (38%) | 9 (23%) |
| P2Y12 Inhibitors (%) | 40 (10%) | 19 (19%) | 5 (8%) | 2 (4%) | 2 (6%) | 4 (5%) | 5 (24%) | 3 (8%) |
| Oral Antidiabetics (%) | 64 (16%) | 33 (33%) | 15 (24%) | 5 (10%) | 2 (6%) | 6 (7%) | 2 (10%) | 1 (3%) |
| Bronchodilators (%) | 26 (7%) | 5 (5%) | 4 (6%) | 2 (4%) | 3 (10%) | 8 (9%) | 2 (10%) | 2 (5%) |

**Additional file 2: Table S1:** Past medical history and medications (abbrev.: AV = aortic valve; MV = mitral valve; LVAD = left-ventricular assist devices).
