# Supplementary material for: Angiopoietin-2 is associated with capillary leak and predicts complications after cardiac surgery
Source: Ann Intensive Care. 2023 Aug 8;13:70. doi: 10.1186/s13613-023-01165-2 (PMC10409979; doi:10.1186/s13613-023-01165-2)
Supplement: Supplementary file 3 — Additional file 3: Table S2. Perioperative echocardiographic metrics. [file 13613_2023_1165_MOESM3_ESM.docx]

**Additional file 3: Table S2:**

| **Echocardiography** | *All patients (N=393)* | *CABG (N=100)* | *AV (N=63)* | *MV (N=52)* | *Multivalve (N=32)* | *Aorta (N=86)* | *LVAD (N=21)* | *Others (N=39)* |
| --- | --- | --- | --- | --- | --- | --- | --- | --- |
| *Left ventricular ejection fraction* |  | | | | | | | |
| LVEF ≥ 55% (%) | 242 (61%) | 48 (48%) | 41 (65%) | 38 (73%) | 24 (75%) | 61 (71%) | 0 | 30 (77%) |
| LVEF 45 – 54% (%) | 62 (16%) | 28 (28%) | 9 (14%) | 4 (8%) | 5 (16%) | 11 (13%) | 0 | 5 (13%) |
| LVEF 30 – 44 % (%) | 38 (10%) | 14 (14%) | 10 (16%) | 6 (12%) | 2 (6%) | 4 (5%) | 0 | 2 (5%) |
| LVEF < 30% (%) | 38 (10%) | 7 (7%) | 3 (5%) | 3 (6%) | 0 | 3 (4%) | 21 (100%) | 1 (3%) |
| *Aortic stenosis (AS)* |  | | | | | | | |
| Mild AS | 8 (2%) | 1 (1%) | 0 | 1 (2%) | 2 (6%) | 3 (4%) | 0 | 1 (3%) |
| Moderate AS | 11 (3%) | 1 (1%) | 4 (6%) | 0 | 1 (3%) | 5 (6%) | 0 | 0 |
| Severe AS | 54 (14%) | 0 | 42 (67%) | 0 | 4 (13%) | 7 (8%) | 0 | 0 |
| *Aortic insufficiency (AI)* |  | | | | | | | |
| Mild AI | 88 (22%) | 16 (16%) | 17 (27%) | 12 (23%) | 5 (16%) | 28 (33%) | 2 (10%) | 8 (21%) |
| Moderate AI | 41 (10%) | 2 (2%) | 7 (11%) | 2 (4%) | 8 (25%) | 18 (21%) | 1 (5%) | 3 (8%) |
| Severe AI | 26 (7%) | 0 | 12 (19%) | 1 (2%) | 2 (6%) | 10 (12%) | 1 (5%) | 0 |
| *Mitral regurgitation (MR)* |  | | | | | | | |
| Mild MR | 115 (29%) | 42 (42%) | 26 (41%) | 2 (4%) | 5 (16%) | 28 (33%) | 3 (14%) | 11 (28%) |
| Moderate MR | 53 (14%) | 14 (14%) | 8 (13%) | 8 (15%) | 5 (16%) | 9 (11%) | 5 (24%) | 4 (10%) |
| Severe MR | 72 (18%) | 1 (1%) | 1 (2%) | 39 (75%) | 22 (69%) | 4 (5%) | 5 (24%) | 0 |
| *Diastolic Dysfunction (%)* | 89 (23%) | 22 (22%) | 20 (32%) | 10 (19%) | 9 (28%) | 16 (19%) | 4 (19%) | 8 (21%) |
| *Systolic Right Ventricular Dysfunction (%)* | 50 (13%) | 8 (8%) | 2 (3%) | 8 (15%) | 6 (19%) | 3 (4%) | 13 (62%) | 10 (26%) |

**Additional file 3: Table S2:** Perioperative echocardiographic metrics.
