# Supplementary material for: Angiopoietin-2 is associated with capillary leak and predicts complications after cardiac surgery
Source: Ann Intensive Care. 2023 Aug 8;13:70. doi: 10.1186/s13613-023-01165-2 (PMC10409979; doi:10.1186/s13613-023-01165-2)
Supplement: Supplementary file 4 — Additional file 4: Table S3. Uni- and multivariable Cox proportional hazards models for time to extubation. Common risk factors were controlled for in the multivariable model. [file 13613_2023_1165_MOESM4_ESM.docx]

**Additional file 4: Table S3:**

|  | **Unadjusted hazard ratio (95% CI)** | ***P*-value** | **Adjusted hazard ratio (95% CI)** | ***P*-value** |
| --- | --- | --- | --- | --- |
| **Angiopoietin-2** | 0.933 (0.913, 0.952) | ***P*<0.001** | 0.939 (0.92, 0.96) | ***P*<0.001** |
| **Age** |  |  | 0.991 (0.983, 0.998) | ***P*=0.009** |
| **CPB time** |  |  | 0.996 (0.994, 0.998) | ***P*<0.001** |
| **Postoperative hyperglycemia** |  |  | 0.997 (0.995, 1) | ***P*=0.043** |
| **P-F-ratio** |  |  | 1 (0.999, 1.001) | *P*=0.84 |

**Additional file 4: Table S3:** Uni- and multivariable Cox proportional hazards models for time to extubation. Common risk factors were controlled for in the multivariable model (Abbrev.: CPB = cardiopulmonary bypass, P-F-ratio = p_a_O_2_ / F_i_O_2_ ratio).
