# Supplementary material for: Angiopoietin-2 is associated with capillary leak and predicts complications after cardiac surgery
Source: Ann Intensive Care. 2023 Aug 8;13:70. doi: 10.1186/s13613-023-01165-2 (PMC10409979; doi:10.1186/s13613-023-01165-2)
Supplement: Supplementary file 5 — Additional file 5: Table S4. Uni- and multivariable models for ICU LOS. A Cox proportional hazards model was used to control for common risk factors for delayed discharge from ICU level of care. [file 13613_2023_1165_MOESM5_ESM.docx]

**Additional file 5: Table S4:**

|  | | **Unadjusted hazard ratio (95% CI)** | ***P*-value** | **Adjusted hazard ratio (95% CI)** | ***P*-value** |
| --- | --- | --- | --- | --- | --- |
| **Angiopoietin-2** | | 0.928 (0.907, 0.949) | ***P*<0.001** | 0.978 (0.952, 1.004) | *P*=0.094 |
| **Surgery type:** | |  |  |  |  |
|  | Aortic valve^#^ |  |  | 1.125 (0.808, 1.568) | *P*=0.485 |
|  | Mitral valve^#^ |  |  | 1.111 (0.781, 1.581) | *P*=0.559 |
|  | Multivalve^#^ |  |  | 0.852 (0.691, 1.51) | *P*=0.916 |
|  | Aortic surgery^#^ |  |  | 1.035 (0.742, 1.444) | *P*=0.84 |
|  | Left ventricular assist devices^#^ |  |  | 0.573 (0.334, 0.982) | ***P*=0.043** |
|  | Others^#^ |  |  | 1.021 (0.691, 1.51) | *P*=0.916 |
| **CPB time** | |  |  | 0.999 (0.996, 1.001) | *P*=0.265 |
| **P-F-ratio** | |  |  | 1.001 (1.001, 1.002) | ***P*=0.001** |
| **Transfusion** | |  |  | 0.592 (0.458, 0.766) | ***P*<0.001** |
| **Invasive ventilation** | |  |  | 0.333 (0.249, 0.446) | ***P*<0.001** |

**Additional file 5: Table S4:** Uni- and multivariable models for ICU LOS. A Cox proportional hazards model was used to control for common risk factors for delayed discharge from ICU level of care (^#^vs. coronary artery bypass grafting; abbrev.: LOS = length of stay, CPB = cardiopulmonary bypass, P-F-ratio = p_a_O_2_ / F_i_O_2_ ratio).
