# Supplementary material for: Angiopoietin-2 is associated with capillary leak and predicts complications after cardiac surgery
Source: Ann Intensive Care. 2023 Aug 8;13:70. doi: 10.1186/s13613-023-01165-2 (PMC10409979; doi:10.1186/s13613-023-01165-2)
Supplement: Supplementary file 6 — Additional file 6: Table S5. Uni- and multivariable logistic regression models for postoperative dependence on vasoactive drugs. The multivariable model was used to control for risk factors for postoperative dependence on vasoactive drugs. [file 13613_2023_1165_MOESM6_ESM.docx]

**Additional file 6: Table S5:**

|  | **Unadjusted odds ratio (95% CI)** | ***P*-value** | **Adjusted odds ratio (95% CI)** | ***P*-value** |
| --- | --- | --- | --- | --- |
| **Angiopoietin-2** | 1.278 (1.196, 1.375) | ***P*<0.001** | 1.208 (1.126, 1.306) | ***P*<0.001** |
| **Age** |  |  | 1.02 (1.001, 1.04) | ***P*=0.041** |
| **Normal LVEF** |  |  | 0.327 (0.194, 0.544) | ***P*<0.001** |
| **Right ventricular dysfunction** |  |  | 3.925 (1.591, 10.881) | ***P*=0.005** |
| **CPB time** |  |  | 1.009 (1.004, 1.014) | ***P*<0.001** |
| **Prior use of ACEi or ARB** |  |  | 0.656 (0.394, 1.081) | *P*=0.101 |
| **Atrial fibrillation** |  |  | 1.147 (0.623, 2.106) | *P*=0.658 |

**Additional file 6: Table S5:** Uni- and multivariable logistic regression models for postoperative dependence on vasoactive drugs. The multivariable model was used to control for risk factors for postoperative dependence on vasoactive drugs (Abbrev.: LVEF = left ventricular ejection fraction, CPB = cardiopulmonary bypass, ACEi = angiotensin converting enzyme inhibitors, ARB = AT_1_ receptor blockers).
