# Supplementary material for: Angiopoietin-2 is associated with capillary leak and predicts complications after cardiac surgery
Source: Ann Intensive Care. 2023 Aug 8;13:70. doi: 10.1186/s13613-023-01165-2 (PMC10409979; doi:10.1186/s13613-023-01165-2)
Supplement: Supplementary file 7 — Additional file 7: Table S6. Uni- and multivariable models for the association of Ang-2 and the P-F-ratio. Generalized estimating equations (GEE) were used to model this association by accounting for repeated measurements on patients. Established confounders were controlled for. In case, patients were not treated invasively, FiO2 was estimated according to the estimated deliverable fraction of oxygen using non-invasive devices. [file 13613_2023_1165_MOESM7_ESM.docx]

**Additional file 7: Table S6:**

|  | **Unadjusted coefficient (95% CI)** | ***P*-value** | **Adjusted coefficient (95% CI)** | ***P*-value** |
| --- | --- | --- | --- | --- |
| **Angiopoietin-2** | -1.149 (-2.283, -0.014) | ***P*=0.047** | -1.007 (-2.139, 0.125) | *P*=0.081 |
| **Age** |  |  | -1.229 (-1.938, -0.52) | ***P*=0.001** |
| **Obesity** |  |  | -54.775 (-74.631, -34.92) | ***P*<0.001** |
| **CPB time** |  |  | -0.208 (-0.39, -0.026) | ***P*=0.025** |
| **Transfusion of more than 10 units of PRBC** |  |  | -11.513 (-60.425, 37.398) | *P*=0.645 |
| **Antidiabetic drugs** |  |  | 6.694 (-13.744, 27.131) | *P*=0.521 |
| **Myocardial infarction** |  |  | -27.815 (-54.601, -1.028) | ***P*=0.042** |
| **Respiratory tract infection** |  |  | -3.583 (-86.656, -79.49) | *P*=0.933 |

**Additional file 7: Table S6:** Uni- and multivariable models for the association of Ang-2 and the P-F-ratio. Generalized estimating equations (GEE) were used to model this association by accounting for repeated measurements on patients. Established confounders were controlled for. In case patients were not treated invasively, F_i_O_2_ was estimated according to the estimated deliverable fraction of oxygen using non-invasive devices (Abbrev.: LVEF = left ventricular ejection fraction, P-F-ratio = p_a_O_2_ / F_i_O_2_ ratio).
