# Supplementary material for: Angiopoietin-2 is associated with capillary leak and predicts complications after cardiac surgery
Source: Ann Intensive Care. 2023 Aug 8;13:70. doi: 10.1186/s13613-023-01165-2 (PMC10409979; doi:10.1186/s13613-023-01165-2)
Supplement: Supplementary file 9 — Additional file 9: Fig. S2. a Standard cross validation, b leave-one-patient-out cross validation, c leave-one-surgery out cross validation. [file 13613_2023_1165_MOESM9_ESM.pdf]

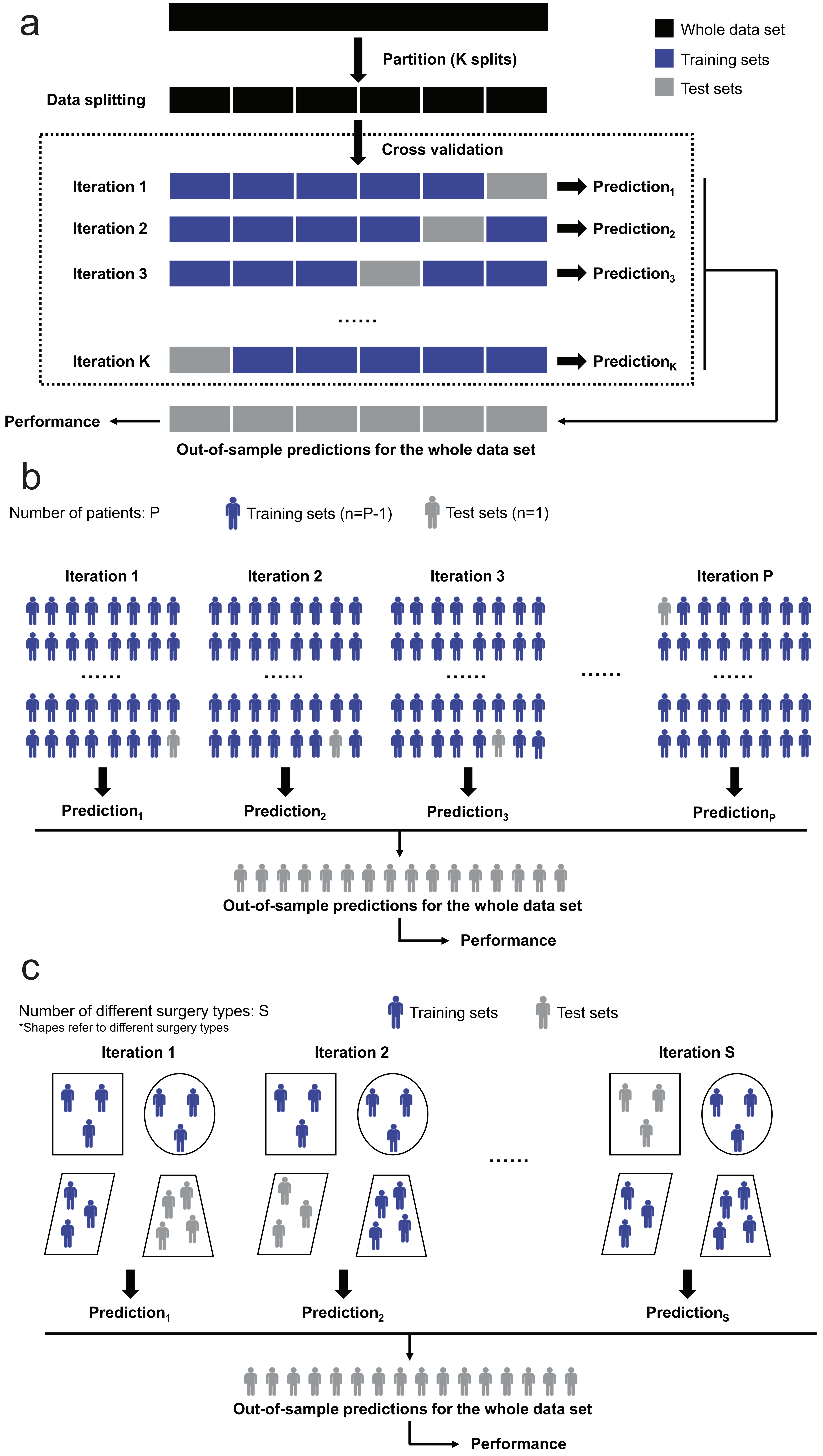
a
Whole data set
Training sets
Test sets
Partition (K splits)
Data splitting
Cross validation
Iteration 1
Iteration 2
Iteration 3
......
Iteration K
Prediction$_1$
Prediction$_2$
Prediction$_3$
Prediction$_K$
Performance
Out-of-sample predictions for the whole data set
b
Number of patients: P
Training sets (n=P-1)
Test sets (n=1)
Iteration 1
Iteration 2
Iteration 3
Iteration P
Prediction$_1$
Prediction$_2$
Prediction$_3$
Prediction$_P$
Out-of-sample predictions for the whole data set
Performance
c
Number of different surgery types: S
*Shapes refer to different surgery types
Training sets
Test sets
Iteration 1
Iteration 2
Iteration S
Prediction$_1$
Prediction$_2$
Prediction$_S$
Out-of-sample predictions for the whole data set
Performance
